# Secret ingredients that can boost the flavor of your ideas

**DOI:** 10.1117/1.NPh.12.4.040101

**Published:** 2025-12-24

**Authors:** Anna Devor

**Affiliations:** Boston University, Department of Biomedical Engineering, Boston, Massachusetts, United States

## Abstract

The editorial reflects on the secret ingredients that can elevate the flavor of our scientific ideas.

How to come up with a good idea? Well, we all know the story! In my version, a woman sits down under a big apple tree, maybe looking for a shady refuge from a late summer sun. An apple falls from the tree, hits our lovely lady in the head and – voila! – she has a brilliant idea!

**Figure:**
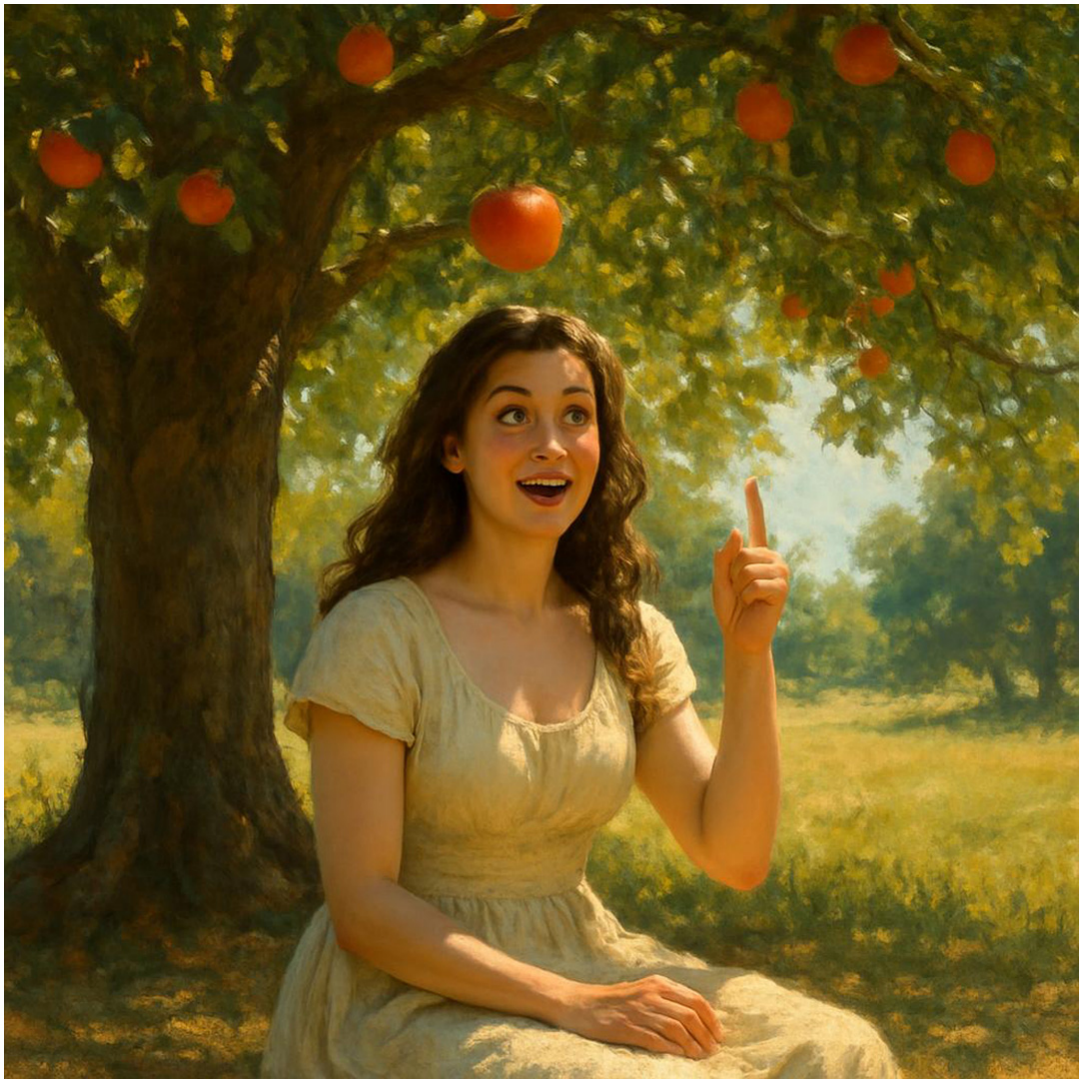
This image was generated by Google Gemini using the first paragraph as a prompt.

To give credit where credit is due, this is a great story. It also has an element of truth in that an external input (in our story, the apple) sometimes can cause the pieces to fall into place revealing a more complete picture. Let’s call this picture a hypothesis. The pieces, however, had to exist in her head in the first place as well as an ongoing mental effort of trying to fit them together in ways that make sense. In other words, there is a process.

When we talk about ideas, we often borrow from the culinary vocabulary. We say, “this is a raw idea” or “this idea is half-baked” or “let’s put this idea on the back burner.” I like that. For me, both science and cooking are forms of art and joys of life. So, in the spirit of the holiday season, let’s talk about some of the secret ingredients that can elevate the flavor of our scientific ideas.

• **#1 – Sparkling mineral water**

Makes brighter colored vegetables, fluffier cakes and waffles, extra crunchy tempura, and festive drinks! If you are a trainee, learning to be creative in your scientific domain is not unlike learning a new language – you have to bubble before you can talk. Relax (flavored soda, anyone?) and embrace trial and error. We’ve all been there.

• **#2 – Cardamon**

Did you ever try surprising yourself and others by adding cardamon to cinnamon rolls? Don’t feel constrained. You are the chef, and this is your personal style restaurant. As a minimum, it will stir up an interesting debate!

• **#3 – Anchovies**

I’m referring to savory, umami-rich items also known as knowledge. This part gets better with years. So, come with me, stand on the shoulders of those who came before us. The horizon is a greater distance away from up here!

• **#4 – Truffle fish sauce**

This is a delightful fusion of Thai and Italian. Blending techniques and flavors from different cultural traditions is fun. It’s also a superpower of the interdisciplinary approach: impactful solutions to difficult scientific problems often arise from the intersection of different perspectives and methodologies.

• **#5 – Lemon zest**

Adds bright, fresh citrus energy to a wide variety of dishes. Keep generating fresh ideas and don’t be shy to bounce them off others. Collaborate with generative AI but also find people who know more than you. Expertise is a wonderful thing.

• **#6 – Fennel seed**

Looking for an extra layer of complexity? It’s not a crime but make sure that the idea is expressed in a quantitative and falsifiable way. And, don’t be disheartened if it does not pass the test. If you are like me, you’ll need to have many bad ideas for a few good ones to emerge.

• **#7 – Coffee**

Adds depth to chocolate desserts. Indulge yourself in the creative process but don’t get too emotionally invested in your baby hypothesis. Save your emotions for those with whom you’ll be enjoying your Chocolate Royal!

As always, I’d like to wish Happy Holidays and Happy New Year to the *Neurophotonics* Editorial Board, our Authors, Reviewers, Readers, and SPIE friends! May the light be with you in 2026!

Anna Devor

*Neurophotonics* Editor-in-Chief

